# Case of a Diseased Prostate Bladder and Rectum

**Published:** 1814-03

**Authors:** Jessé Foot

**Affiliations:** Surgeon


*For the Medical and Physical Journal.*

### *Case of a diseased Prostate Bladder and Rectum; by* Mr. Jessé Foot, Surgeon.

IN October, 1818, a person came to me from Bromley in Kent, to take my advice on his complaints; and as he had already taken up his abode at a friend's in Bedfordsquare, for that intent, we devoted this first visit to a conversation on the nature of his symptoms, the time they had been on him, the remedies which had been used, the medical gentlemen under whose care he had been, and on the whole of that information which can be better collected from a patient himself, than from any body else.

He was at the advanced age of sixty, of an uncommon calmness of disposition for one so terribly afflicted and so very much emaciated. He was a married man, of very sober and regular habits, had been first attacked a year and a half ago with a disorder in his bowels, for which he had from the beginning consulted an eminent physician of the Borough hospitals, and whose prescriptions he brought with him. He had also consulted a surgeon of St. George's hospital, and a surgeon of the Borough hospitals, some time before he applied to me.

Doubts having arisen that the disease was a prostate case, which was the opinion of the surgeon of St. George's hospital, he consulted the surgeon of the Borough hospitals, who sounded his bladder in search of stone; but none being found, and the case proceeding from bad to worse, in the face of all the remedies hitherto administered, it was at length decided that he labored under an incurable prostate case; and the patient was viewed in the light of one condemned to the excesses of suffering, so long as life supported him in the endurance.

Having heard attentively that evening all he had to say, I promised him that the following morning I would satisfy myself, by an examination into his case in the fullest manner I possibly could. He accordingly came, and gave me his report of the night he had passed: that from eleven at night to nine in the morning he had urined ten times; and that he had also made many attempts without being able to urine at all, especially when he wanted to go to stool, which was also as distressing to him as the act of urining, as he would have the solicitation to stool for two, and oftentimes three hours, before any stool could pass. He got out of bed every time he urined, or tried to urine.

I examined the prostate; it was enlarged and fixed. I passed a gum catheter of a middle size; it went readily into the bladder. The urine was drawn off, and measured by a scale cut on a glass cup two ounces. This was the largest quantity he was in the habit of discharging at any one time. The first half of the urine came away clear, the other was of that turbid appearance which resembles chalk mixed up with thin mucilage; and this of course was all that the bladder could hold. The sphincter ani was found to be very rigid, rigid. I filled the bladder with warm water by injection. It could not bear more than two ounces and a half, and that extension of the bladder produced great pain. The catheter I chose was of an extraordinary length, and I was enabled to pass it to the *fundus* of the bladder, which I conceived to be so contracted as not to admit of containing, by any effort of extension, more than two ounces and a half. The circumstance of the turbid urine, as well as the clear urine, coming away through the catheter, and of the turbid urine being the last that passed, proved to me that this was a disease of the bladder and prostate, and that the prostate was not the part only diseased. When the whole contents of the bladder were drawn off, the pain the patient expressed was exquisite. He could neither sit nor stand quiet for some time. On the evening the bladder was filled with warm water again, and it was again drawn off to much the same amount as in the morning. The filling the bladder with warm water was from henceforth repeated daily, twice a day. The additional resources were sitting night and morning on a bidet filled with hot decoction of poppies, and on the second night eight leeches were applied to the perineum. His bowels were relieved by castor oil taken over night, but there was always a difficulty in procuring an evacuation. The time it occupied from the first peristaltic inclination to that of the evacuation would include the best part of the night, during which he experienced a more than ordinary difficulty of urining. He described himself as having lost the power and assistance of the abdominal muscles, and I found that the *acceleratores urinæ* and *sphincter ani* were strongly affected by their increased efforts through such a protracted time of disease. Finding that by the prescriptions mercury had been given to the patient for some considerable time, I from that cause gave him none. I had promised him, notwithstanding the pain he felt, that if he would submit to try the extension of the bladder for *three weeks*, and if then he was not better I should give up the pursuit. I therefore thus continued the process, when for the first four days there appeared but little variation from the beginning, neither in quantity, appearance of the urine, nor in any other symptom. But it was rather thought that his days were better, but not his nights, which was the time his stools were disposed to pass, and which was very distressing to him. In the subsequent four days, the bladder became so far dilated as to receive nearly four ounces of warm water, and the solicitation to urine in the day-time was somewhat abated. But still the difficulty of procuring stools distressed him throughout the night, and shortened his intervals of urining.

Before a fortnight had passed, the bladder was brought to contain six ounces of warm water. A new complaint then arose: his right testicle was swelled, but not accompanied with so much pain as it had been when swoln before, after he had consulted the surgeon of St. George's hospital, when it confined him to his bed a month. By giving it support, and applying compresses dipped in *Aq. Ammon. Acetat.* this complaint disappeared in four days; for we know, that a testicle once having swelled, does not give the pain a second time in swelling, and that it is with much less difficulty reduced. During this interval I desisted from injecting the bladder, but renewed it on the fifth day. I now for the first time began to give him the *Uva Ursi* in liberal doses. Finding that the difficulty of obtaining stools was a continued theme of complaint, I began to question him very narrowly upon this part of the case; and it appeared from his answers that his bowels had been disordered three months before his difficulty in urining came on; that he did not recollect at any time that he had, or was capable of discharging a figured stool of natural dimension, such as confirms the bowels to be in a sound state. Besides, he answered, that, although he had often taken clysters, they were generally returned without fæces. I passed the largest urethra bougie I had, and found an obstruction in the *rectum*, about five inches from the *anus*. Small rectum bougies were from this time daily passed, gradually increasing their sizes. This he adroitly did himself. From this time his stools began evidently to be more compact, more ready in coming away, and more easy in their evacuation. This complaint of his bowels had been on him more than eighteen months, as his prescriptions for it proved, by their dates.

Towards the latter end of the third week, his recovery was no longer a matter of doubt. Seven ounces and a half of warm water could be injected with ease, and drawn off without pain. The urine began to change from the wheyish appearance it had to that of a sound state. Nothing new was done for the case from this time; nothing further but repeating the injection of warm water twice a day, passing the rectum bougies, taking the *Uva Ursi*, and sitting over the decoction of poppies. At the close of the fourth week, the bladder could contain eleven ounces, and the patient could sit throughout the night at the theatre, without a call to urine. He then made a trip to Bromley, and returned a week after, when, for the last time, his bladder was injected with warm water, and eleven ounces and a half returned through the catheter. At this period he urined at healthful distances, and his stools passed with lessened irritation, and more more approaching to a condition of health. When he had expended the graduated sizes of rectum bougies I had furnished him with, I recommended him to have some made of *spermaceti*, of increased sizes, and of proper length. I saw the patient in the middle of January, when he described himself to be well.

In this case I have not gone into the theory of the diseases of prostate and bladder, and of the advantages of *Vesicæ Lotura* in expanding a contracted bladder, because I have done this already in a former publication of cases of the successful practice of Vesicæ Lotura.

It has been shown by the relation which I have given, that this case was by me difficult to be defined; that two hours attention to it daily, did not unfold to me a knowledge of its true nature, until a fortnight had passed. I am afraid I may be censured for want of that ready intuition which some practitioners possess, who limit their visits to about five minutes, and others who correspond by letters upon cases; but I must confess, by either of these methods I should never have discovered that which in this extraordinary case has contributed towards so rapid a removal of it.

*Dean-street, Soho,* JESSE FOOT.
*Feb.* 21, 1814.

